# “From taboo to routine”: a qualitative evaluation of a hospital-based advocacy intervention for domestic violence and abuse

**DOI:** 10.1186/s12913-020-4924-1

**Published:** 2020-02-21

**Authors:** Sandi Dheensa, Gemma Halliwell, Jennifer Daw, Sue K. Jones, Gene Feder

**Affiliations:** 1 Domestic Violence/Abuse and Health Research Group (DVAHG), Centre for Academic Primary Care, Bristol Medical School, University of Bristol, Canynge Hall, 39 Whatley Road, Bristol, BS8 2PS UK; 2 Safelives, Suite 2a, Whitefriars, Lewins Mead, Bristol, BS1 2NT UK

**Keywords:** Domestic violence, Intimate partner violence, Advocacy, Midwifery, Health personnel, Emergency medicine, Health, Health services

## Abstract

**Background:**

Health services are often the first point of professional contact for people who have experienced domestic violence and abuse. We report on the evaluation of a multi-site, hospital-based advocacy intervention for survivors of domestic violence and abuse. Independent Domestic Violence Advisors (IDVAs), who provide survivors with support around safety, criminal justice, and health and wellbeing, were located in five hospitals in England between 2012 and 2015 in emergency departments and maternity services. We present views about IDVAs’ approaches to tackling domestic violence and abuse, how the IDVA service worked in practice, and factors that hindered and facilitated engagement with survivors.

**Methods:**

We adopted a convenience sampling approach and invited participation from all who offered to take part within the study timeframe. Sixty-four healthcare professionals, IDVAs, IDVA service managers, and commissioners at all sites were interviewed. Interviews were analysed using a thematic approach: familiarising ourselves with the data through repeated readings and noting initial ideas; generating initial codes through double coding notable features of the data across the dataset; collating codes into potential themes; and reviewing themes to ensure they captured the essence of the data.

**Results:**

Two key themes emerged. The first was *Hospital-based IDVAs fulfil several crucial roles.* This theme highlighted that healthcare professionals thought the hospital-based IDVA service was valuable because it enhanced their skills, knowledge, and confidence in asking about domestic violence and abuse. It enabled them to immediately refer and provide support to patients who might have otherwise been lost along a referral pathway. It also reached survivors who might otherwise have remained hidden. The second theme was *Success hinges on a range of structural factors*. This theme illustrated the importance of ongoing domestic violence and abuse training for staff, the IDVA having private and dedicated space, and the service being embedded in hospital infrastructure (e.g. featuring it in hospital-wide policies and enabling IDVAs access to medical records).

**Conclusion:**

Hospital-based IDVAs offer a unique and valued way to respond to domestic violence and abuse in a healthcare setting. Further work must now be done to explore how to implement the service sustainably.

## Background

An estimated two million adults in England and Wales aged 16 to 59 experienced domestic violence and abuse in the year ending March 2018, two-thirds of whom were women [1]. The health consequences of domestic violence and abuse are wide-ranging, as Table 1 from the World Health Organization [2] illustrates. In the UK, the National Health Service (NHS) is often the first point of professional contact for survivors [3]. Healthcare professionals are well-placed to respond to domestic violence and abuse.

**Table 1:** Health consequences of domestic violence and abuse (World Health Organization)

**Physical**	**Sexual and reproductive**
- Acute or immediate physical injuries, such as bruises, abrasions, lacerations, punctures, burns, and bites, as well as fractures and broken bones or teeth	- Unintended/unwanted pregnancy
	- Abortion/unsafe abortion
- More serious injuries, which can lead to disabilities, including injuries to the head, eyes, ears, chest, and abdomen	- Sexually transmitted infections, including HIV
	- Pregnancy complications and miscarriage
- Gastrointestinal conditions, long-term health problems, and poor health status, including chronic pain syndromes.	- Vaginal bleeding or infections
	- Chronic pelvic pain
- Death, including femicide and AIDS-related death	- Urinary tract infections
	- Fistula (a tear between the vagina and bladder, rectum, or both)
	- Painful sexual intercourse
	- Sexual dysfunction
**Mental**	**Behavioural**
- Depression	- Harmful alcohol and substance use
- Sleeping and eating disorders	- Multiple sexual partners
- Stress and anxiety disorders (e.g. post-traumatic stress disorder)	- Lower rates of contraceptive and condom use
- Self-harm and suicide attempts	
- Poor self-esteem	

The UK’s National Institute for Health and Care Excellence [4] recommends that NHS staff should be trained to identify and respond to domestic violence and abuse and to refer survivors to specialist services. However, training varies across the UK and across different clinical specialties.

Various services and interventions exist in the UK that aim to increase and improve identification and responses to domestic violence and abuse in the healthcare setting. One NHS Trust for example has developed a domestic abuse nurse specialist role. The nurse develops and delivers staff training and resources, supports clinical staff with assessing and referring survivors, supports survivors, and streamlines referral pathways to external agencies and specialist services [5–7].

Complex interventions have also been trialled and implemented in the UK. Several interventions, based in midwifery [8–11] (because pregnancy is a high-risk time [12]) and primary care [13, 14], have involved training for healthcare professionals and implementing policies on asking patients about domestic violence and abuse and explicit referral pathways to services. Healthcare professionals have been satisfied with such training and it increases healthcare professional confidence and knowledge [11, 15]. Patients have valued improved referral pathways and contact with domestic violence and abuse services that follows quickly after a healthcare professional’s referral [16].

A smaller group of interventions has involved co-locating Independent Domestic Violence Advisors (IDVAs) in hospital departments, in conjunction with training healthcare professionals and implementing policies and referral pathways. IDVAs provide survivors of domestic violence and abuse with support around criminal justice, housing, health and wellbeing, finances, employment, and immigration. They represent survivors’ voices at multi-agency fora and work in partnership with agencies to assess risk and develop co-ordinated safety plans for survivors and their children [17–19]. Research shows that intensity of support (e.g., number of contacts, longer case length) and access to community resources and/or interventions through community-based advocacy services, including through IDVAs, is associated with an increased sense of safety and cessation of abuse for survivors [20]. Advocacy may improve quality of life and reduces physical abuse in the short term. Limited and emerging evidence shows it can improve mental health [21].

Three studies have evaluated the co-located IDVA model. One such intervention was based in a hospital’s gynaecology, HIV medicine, and genitourinary medicine clinics [22]. The IDVA here also dealt with sexual violence and was available during business hours to see referred patients and to advise, train, and support staff. Staff implemented routine inquiry (asking all patients about domestic violence and abuse). Of 10,158 patients asked, 718 (7.1%) reported ever experiencing it. Staff made 77 referrals to the IDVA. Survivors had higher rates of previous emergency department attendances, emergency inpatient admissions, and day-case admissions than patients who had not experienced domestic violence and abuse. Compared with the IDVA’s general (non-hospital) referrals, hospital referrals were more likely to be classified as facing high-risk and were more likely to accept support.

Two other IDVA interventions have been based in emergency departments. In one [23], mandatory training was provided to senior staff for a year, who then adopted clinical enquiry (asking patients when there is suspicion of domestic violence and abuse). A screening tool, referral pathway to a domestic violence and abuse service, and electronic coding system were also developed. In total, staff referred 121 patients. IDVAs reported that they had developed a good working relationship with staff. Staff said that they were more aware of domestic violence and abuse following the intervention, clearer on what to do in suspected cases, and more comfortable with enquiring. In another intervention, *REACH* [24], healthcare professionals saw the IDVAs’ placement as an opportunity to provide immediate crisis intervention, to identify survivors who might not seek help from other sources, and to relieve emergency department staff from managing disclosures.

Interventions such as these can help to address some of the barriers that healthcare professionals face to enquiring about domestic violence and abuse—e.g. reluctance to ask because they do not know how to manage disclosures and a lack of confidence and knowledge about referral pathways or available support [5–7, 13, 25, 26]. However, obstacles arise in IDVA interventions. For example, emergency department IDVAs in the *REACH* [24] intervention found it difficult to see patients alone in a private space and faced bureaucratic hurdles, such as having several managers and no access to emails. These hurdles hindered them from feeling like part of the care team.

The current paper reports on the largest IDVA evaluation to date. It presents part of a service evaluation that explored the profile of survivors, the work of hospital-based IDVAs, survivors’ outcomes, and facilitators and barriers to basing domestic violence and abuse services in hospitals [27, 28]. This paper focuses on this last aspect and presents the qualitative component of the evaluation, drawn from interviews with hospital staff, domestic violence and abuse service staff, and commissioners.

### Multi-site hospital-based IDVA intervention

Between 2012 and 2015, five hospitals across England (one large city, one medium-sized city, and three smaller towns) launched an integrated advocacy approach to supporting domestic violence and abuse survivors in a hospital setting, whereby IDVAs were co-located in emergency department and maternity services. IDVAs could also refer survivors directly to mental health services. Safelives, a UK domestic abuse charity, led on evaluating the project. The quantitative aspect of the evaluation is published [27].

The IDVAs’ roles were to provide immediate support and advice to domestic violence and abuse survivors within the hospitals; to link individuals and families to longer-term community-based support; and to train hospital staff to increase confidence in asking about domestic violence and abuse. Training content varied across different sites according to need, but broadly covered the dynamics of domestic violence and abuse; the relationship with drug and alcohol use, mental health, disability, age, and pregnancy; medical signs and symptoms; how to effectively ask about domestic violence and abuse; and information-sharing and confidentiality guidelines for high-risk cases or cases with adult or child safeguarding concerns. Each IDVA worked at the hospital at different times. Table 2 illustrates two examples in more detail, including hospital type, number of staff, patient load, the IDVA service age, cost, funder, and the IDVA’s employer. The annual cost to run the service was £90,000 for the site with 7000 staff and £40,720 for the site with 3000 staff. The annual number of patient referrals to the IDVA was 365 in the former and 97 in the latter. The evaluation reached 692 survivors across 3 years, although the services will have since reached many more [27, 28].

**Table 2:** IDVA service case studies

	Case 1	Case 2
Hospital type	Large metropolitan hospital	Smaller rural hospital
Number of staff	7000	3000
ED patient-load	70 k/annum	42 k/annum
Age of service	5 years old	3 years old
Service cost 2014–15	£90,000	£40,720
Funded by	NHS England, Local Clinical Commissioning Group, City Council Public Health	Primary Care Trust initially, then a charitable trust
IDVAs employed by	Hospital trust	Third sector domestic violence and abuse organisation
Institutional integration	Full - staff are Trust employees with NHS badges, access to NHS emails and hospital computer system, able to ‘flag and tag’ cases and receive real-time alerts when patients with a history of domestic violence and abuse attend the emergency department.	High – IDVAs have an honorary NHS contract, enabling them to have an NHS badge, access to NHS emails and ability to ‘flag and tag’ cases on the hospital computer system. However, second IDVA faced six month delay getting contract.
Visibility	Very high – based in a room in the emergency department, IDVAs regularly use staff room	Very high - based in a room outside the main hospital building, but IDVA visited the emergency department and maternity wards regularly and could see patients in a quiet room in both locations.
Publicity	Posters widespread in hospital – plus use of other materials (e.g. mouse mats)	Leaflets and posters (after approval by six panels).
Number of IDVAs	Two full-time, seven days a week 9 am–5 pm	One full-time equivalent (two job-sharing), Monday to Friday 9 am-5 pm
Number of HCPs trained	271 in 2014–15	200 (plus 35 General Practitioners); 120 in 2015–16 (plus 27 General Practitioners)
Number of referrals 2014–15	365	97
Referral method	Often face-to-face by calling into IDVAs’ room, by phone, or (out-of-hours) by online referral form (including risk assessment) supplemented by access to the patient’s online hospital notes.	Emergency department staff mostly used paper forms; psychiatric liaison mostly used phone during office hours; maternity mostly used phone or told IDVA face-to-face on her regular ward visits.

## Method

This was a qualitative study using semi-structured interviews with professionals. South-West Central Bristol Research Ethics Committee approved the study (13/SW/0012). We developed interview schedules, with key open-ended questions and probes, to guide our interviews. The Safelives research team (including co-authors Daw and Jones) developed these between 2014 and 2015 based on a literature review about identifying and responding to domestic violence and abuse in the health setting and on preliminary conversations with domestic violence and abuse service staff based in hospitals. Safelives’ survivor and expert panels (which meet regularly) reviewed the schedules.

We recruited our sample through our co-investigators (one was based at each site). Potential participants received information sheets stating the research aims and could take part by phoning or emailing the research team or by stating their interest to co-investigators or researchers face-to-face. The information sheet informed them that they could withdraw from the study at any time without giving reason. All participants gave written consent to take part. The main ethical issues were that discussion of a sensitive topic area could upset participants and cause ‘vicarious trauma’ to the research team. To mitigate these risks, participants were offered contact details of local support services and researchers were offered clinical supervision.

We adopted a convenience sampling approach and gave the opportunity to participate to all those who offered to take part within the study timeframe. Co-author Jones conducted all interviews in 2015 at the sites and made field notes during interviews. She had no prior relationship with the participants. Interviews were between 20 and 75 min long. Given the shortness of some interviews and our desire to capture a range of voices –i.e. healthcare professionals from different specialties, we interviewed 64 participants across sites: forty-nine hospital staff, six hospital IDVAs, four IDVA managers, and five commissioners. Table 3 contains a breakdown of roles. We did not record numbers of people who refused to participate.

**Table 3:** Participants’ roles

Roles	Number of interviews
Hospital staff	
- Emergency medicine consultants	7
- Emergency medicine junior doctors/ house officers	3
- Emergency medicine nurses or sisters	12
- Safeguarding children or adults named nurses	6
- Psychiatrists	3
- Mental health nurses	8
- Alcohol and drug nurses	1
- Midwives and midwife managers	6
- Other medical staff	1
- Research and human resources staff	2
- Sub-total	49
Other staff	
- IDVAs	6
- Commissioners	5
- IDVAs’ managers	4
- Sub-total	15
TOTAL	64

**Fig. 1:**
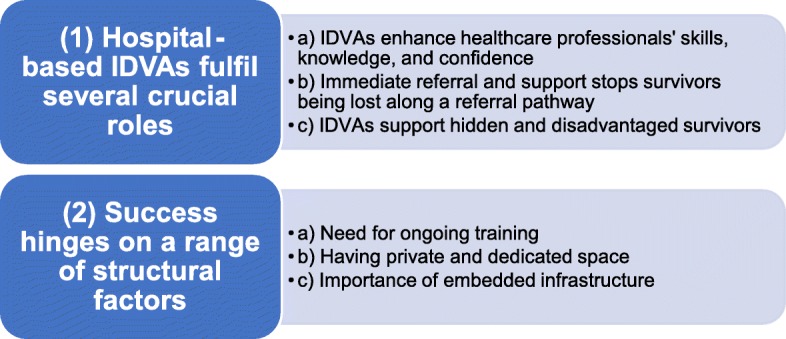
Summary of themes

Over the course of our interviews, our interview schedules evolved so that we deprioritised questions about how healthcare professionals generally respond to domestic violence and abuse and prioritised questions about their views about the co-located IDVA service. We adopted thematic analysis as our analytic approach [29] and began analysing data in tandem with data collection. Once we began our analysis, we added and amended our interview schedule to interrogate emergent themes. Three researchers, Dheensa, Daw, and a Safelives data support officer, analysed the data. All of us are women researchers with social science and health science postgraduate research qualifications. We derived our themes from the data following the iterative phases of our thematic analysis approach [29]. Daw and the data support officer led on familiarising themselves with the data through repeated readings and noting initial ideas; generating initial codes through double-coding notable features of the data across the dataset; and collating codes into potential themes. Dheensa led on reviewing themes to check that they captured the essence of the coded extracts and the entire dataset and defining and naming themes. Jones stopped interviewing upon approaching saturation of the main topics. To give this paper focus, we have chosen to exclude data about healthcare professionals’ general views about domestic violence and abuse and how they might respond to it without an IDVA service. Figure 1 shows our two main themes and subthemes.

## Results

### Hospital-based IDVAs fulfil several crucial roles

Hospital-based IDVAs fulfilled various functions that potentially would have gone unfulfilled in the hospital setting. The subthemes below illustrate this finding.

#### IDVAs enhance healthcare professionals’ skills, knowledge, and confidence

Healthcare professionals considered the IDVA service to be “incredible” (Location 1, Emergency Department Lead Nurse) and “integral” (Location 1, Adult Safeguarding Nurse) to the hospital. They felt that the training that IDVAs delivered helped them to ask patients questions about domestic violence and abuse more effectively and sensitively and to detect subtler signs of domestic violence and abuse.

“Asking around the issue, you get a sense of their world. Gaining someone’s trust and showing interest. You go from that, ‘I am cold at home’, to, ‘I’m not allowed to put the heating on’, to, ‘because John won’t let me'.” (Location 3, Practice Development Nurse)

“You are not putting words in their mouth but empowering them to say it. ‘We have seen these injuries that have been based on domestic violence in the past. Is there anything you would like to tell me?’ A lot of that has come from our IDVA here: from taboo to routine.” (Location 1, Emergency Department Consultant)

Healthcare professionals particularly valued the fact that IDVAs signposted them to further training opportunities outside of the core training, such as training on responding to male survivors.

As well as benefitting from this initial and ongoing training, healthcare professionals felt they benefitted from the IDVA’s co-location. Given the difficulties broaching a sensitive topic in a busy environment, healthcare professionals felt co-location made practice more efficient when they were faced with a patient in need of support:

“It’s a complex and emotive subject [so] it is really nice to have a person when we know we have concerns … sometimes we have a hunch and we have somebody to say, ‘Can I just run it past you?’” (Location 2, Hospital Midwife)

Healthcare professionals and commissioners said that referring to IDVAs saved time, and so despite the costs associated with setting up the IDVA service, it was economically beneficial to the health service:

“It’s really helpful to have input from IDVA … [previously] one of my nurses spent a whole day and I spent a whole afternoon trying to find one refuge.” (Location 4, Consultant Psychiatrist)

“We can extrapolate the money saved by the hospital IDVA service. ‘Spend to save’ agenda.” (Commissioner)

#### Immediate referral and support stops survivors being lost along a referral pathway

Interviewees valued the intervention because it meant healthcare professionals could directly refer survivors for support. Although other healthcare-based domestic violence and abuse interventions (e.g., [13]) also offered direct referral, the IDVA’s co-location had the added benefit that survivors could receive support immediately. These two benefits allayed healthcare professionals’ worries, which they based on previous encounters, that survivors might disclose domestic violence and abuse but ‘disengage’ before receiving support; that survivors might later recant their disclosure through fear of repercussions; or that healthcare professionals would have nothing to offer them following a disclosure. These worries reflect how difficult it can be to get a survivor to safety and the below quotes illustrate the value of direct referral:

“[Staff] wanted to feel they could do something there and then about it. Not ‘thank you for telling me. Here’s the information. Call them without me.’ You could lose them.” (Location 3, Senior Sister)

“Having the IDVA means we have a clear pathway of referral which is important. There’s a big difference between identifying abuse and knowing there is something they can do.” (Location 4, Emergency Department Consultant)

The IDVA quoted below reiterates this point: the offer of direct referral and immediate support was crucial for enabling healthcare professionals to feel more confident that enquiring about domestic violence and abuse was worthwhile:

"Knowing that we are on site [is beneficial]. A lot of practitioners are worried about disclosures. 'We have opened a can of worms. What can we offer?'" (Hospital IDVA)

Direct referral and immediate support was important also because healthcare professionals often saw survivors at a unique time: when they were physically injured to the point of needing hospital care or when they were in a mental health crisis (e.g. with “psychiatric presentations, overdoses” (Emergency Department consultant)). healthcare professionals felt that the seriousness of the situation might make survivors more likely than usual to seek support. It was a rare ‘window of opportunity’ that healthcare professionals wanted to seize and as the below quote illustrates, co-location made this seizing more possible:

“We are catching people at point of crisis, at the time. Otherwise they have gone home and been reluctant to engage. We are getting there earlier.” (Hospital IDVA)

“We should have [IDVAs] here as much as we are here. When someone starts to open up, we really want to hit right there and then and say, ‘we’ve got specialist people here to give really good advice, options, and pathways.’” (Location 1, Clinical Nurse Specialist)

Co-location moreover meant that IDVAs could more promptly refer to other hospital-based services, e.g. mental health, hospital-based child protection, and other wards:

“There is a lot more liaison with other specialists, which can be harder to do in community-based service – we are in the same building.” (Senior IDVA)

Thus, according to interviewees, co-location of IDVAs facilitated more holistic, multi-disciplinary, and integrated support.

#### IDVAs support hidden and disadvantaged survivors

Hospital-based IDVAs reported that they saw survivors at an earlier point in the abusive relationship than their clients from the community—which has been found to be the case in other healthcare-based interventions [13]. They also reported that they saw survivors “who are hidden from society” (Senior IDVA)—including from community-based services. Thus, findings indicate that community-based domestic violence and abuse services (with no integration with healthcare) are insufficient alone:

“You get people ‘admitting’ to violence at home after two years in maternity, whereas it’s more than four years in the community.” (Commissioner)

Hidden survivors included men, older survivors, and survivors from higher-income households:

“I saw lot of very wealthy middle-class women who suffered terrible domestic abuse from their husbands.” (Location 3, Matron of Emergency Department)

IDVAs noted that compared with their community caseload, survivors they saw in hospitals seemed to have more complex needs, especially mental health problems:

“Hospital clients – I think their needs are higher because they come in with overdose, attempted suicide, injuries, or alcohol-related issues.” (Senior IDVA)

“[We see] different kinds of clients, for example people with addictions, … A lot have mental health problems. A lot have personality disorders.” (Hospital IDVA)

Survivors with ‘complex needs’, or who face multiple disadvantages—i.e. substance/alcohol use disorder, homelessness, and/or mental ill health—can face numerous barriers to accessing support, such as a lack of suitable services [22]. Healthcare professionals felt that hidden survivors might become visible in a hospital because they see it as “a place of safety and confidentiality” (Emergency Department doctor). They might feel more comfortable talking about domestic violence and abuse and associated issues, such as addictions, in a hospital than in a community service.

Interviewees said that healthcare professionals were in a unique position. They could see patients repeatedly and were able to notice subtler and health-related signs of victimhood, such as "suspicious injuries that don't fit the [presenting condition]" (Emergency Department consultant), as well as seeing how the survivor interacts with, or talks about, the perpetrator. As such, survivors who would usually remain hidden to services and agencies could become visible to healthcare professionals:

“You see frequent attenders with chronic pain, psychiatric presentations, overdoses, almost fictitious disorders … a passport to see the doctor. It’s very rarely about woman turning up missing couple of teeth and big black eye.” (Location 4, Emergency Department Consultant)

“Often you see injuries or aches and pains that don’t necessarily correlate with the patient’s complaints.” (Location 3, Senior Emergency Department House Officer)

“Their mental state suddenly changes if talking about partnership: tearfulness or fear.” (Location 2, Psychiatrist)

“Mum will sit quite a long way back. Father is telling you all about the child. Mother doesn’t speak. You very rarely see any physical evidence, usually more emotional, and body language. And you can see how the child is reacting with parents.” (Location 3, Paediatric Lead Sister)

Having the IDVA on hand meant these hidden survivors could quickly access support.

### Success hinges on a range of structural factors

Interviewees considered various structural factors to be crucial for the implementation of the IDVA service. The subthemes below describe these factors: ongoing training given high staff turnover, the need for physical space for the IDVA, and processes for embedding the service in hospital policies and procedures.

#### Need for ongoing training

Regarding the training aspect of IDVAs’ roles, interviewees were concerned that raising awareness of the IDVA service and changing attitudes and practices among healthcare professionals would be difficult because of the NHS’s huge and disparate workforce, and the constant turnover of staff:

“It is like painting the Forth Bridge.” (Commissioner).

“Training people to understand the importance of asking questions needs constant work and structure.” (Location 3, Team Leader Mental Health)

Healthcare professionals pointed out that training emergency department staff would be particularly tricky, both because of turnover and the number of potential survivors in their patient-load.

Interviewees were especially worried about training in larger organisations, where, as IDVAs pointed out, not all departments would know about the service:

“The sheer scale of the place. All the different wards knowing about us and how to refer to us … I think we are missing quite a lot of opportunities.” (Hospital IDVA)

To tackle this issue, healthcare professionals suggested embedding domestic violence and abuse training in medical education and in postgraduate training and targeting junior doctors:

“If you get the juniors whilst most relevant to them, that would stick in their memory. Then you are training whole cohort of doctors when juniors.” (Location 1, Junior Doctor)

#### Having private and dedicated space

IDVAs and healthcare professionals also emphasised that for the IDVA service to be effective, the IDVA would not only need to be co-located in the hospital, but be a visible and integrated part of the healthcare team with a dedicated physical space such as an office. Visibility helped healthcare professionals see the IDVA service as integral to everyday practice and reminded healthcare professionals to ask about domestic violence and abuse and to refer patients—important given the sometimes chaotic and often under-resourced nature of their clinical departments:

“Domestic violence is in your mind because we walk past their door. Having them here is a constant reminder to us.” (Location 1, Emergency Department Consultant)

Visibility also made healthcare professionals feel more at ease approaching IDVAs, which in turn helped to build relationships between healthcare professionals and IDVAs and improve information-sharing:

“They have lunch in the staff room. They socialise with the team. That is where the success really comes from. They are not seen as a separate and aloof service that we just refer to.” (Location 1, Emergency Department Nurse)

As one IDVA recounted, visibility also made it more likely that patients would know about the service and ask for a referral:

“We would get multiple clients turn up numerous times in [the emergency department], just to see us. They would walk into reception and say, ‘I’m here to see [service name]’.” (Senior IDVA)

Several IDVAs said they had no permanent physical base, perhaps reflecting the under-resourced nature of clinical departments. IDVAs said that this lack of physical base meant opportunities to build relationships were fewer. It led to some feeling like an outsider to the cultures and subcultures of clinical teams, which in turn affected their morale—as well as their ability to see patients:

“I felt really lonely just being there in the beginning. Trying to find people to introduce myself to. It still is lonely.” (Hospital IDVA)

“All of them want me to be there more often – to be visible. But I can’t just loiter. I can’t really see anybody here. I’ve not got a private room to see people in. If I had an office, that might help.” (Hospital IDVA)

As some healthcare professionals pointed out, a lack of privacy increased the risk of confidentiality breaches. However, there was a careful balance to be struck between visibility to healthcare professionals and patients and visibility to perpetrators. Being too visible could lead to retaliation from perpetrators (e.g. if they had accompanied the survivor to hospital) or could lead to other patient companions telling the perpetrator that suspicion of domestic violence and abuse has arisen. IDVAs needed a private *and discreet* space with tactics for separating survivors from perpetrators:

“If word gets out that we are running the [service] and a relative or friend sees the IDVA with the client, it can put the client—and the service—at risk. [We] don’t want too much publicity.” (Location 2, Research Nurse)

“[IDVAs] were initially based here. Part of the problem was they were trackable and traceable. They went off-site for their own security because of perpetrators.” (Location 3. Consultant Emergency Medicine)

#### Importance of embedded infrastructure

As well as visibility and a physical space, healthcare professionals pointed out the need for forward-planning, engagement with staff, and a strategic plan to ensure the service was used:

“The IDVA can be lone voice in massive organisation. For any new IDVA, going into any hospital, there has to be a plan … you have got to sell yourself … to get across what you are there for, in an easy-to-understand way.” (Location 5, Adult Safeguard Lead)

Healthcare professionals pointed out several strategies that were helpful for making the IDVA service known. One was for senior healthcare professionals to champion the service and promote strategic plans:

"For a hospital IDVA service to run properly and be accepted by hospital staff, you need a medical champion. The higher up the better. Junior doctors want to impress them—they don't want to miss stuff. So, if the senior medic says this is important, then they'll look for it." (Senior IDVA)

A second strategy was for IDVAs and healthcare professionals to be able to ‘flag’ patients facing risk of domestic violence and abuse in medical records:

“In past jobs we haven’t had info because people have been anonymous, talking to us on the phone. Here, if people don’t want to engage, we can flag to the hospital and GP [General Practitioner] without consent and feel we are more effective really.” (Hospital IDVA)

However, IDVAs were sometimes unable to do such flagging because it required them to be granted ‘honorary contracts’ with the NHS in order to access patient identifiable data—a time-consuming and bureaucratic process:

“They [another hospital] can put flags on victim records. Here, unless there is a [safeguarding issue] they wouldn’t be able to do that... [Not being] on the system makes it a lot more difficult for partnership working.” (Hospital Services Manager)

When flags were used, processes were more efficient. IDVAs and healthcare professionals could regularly meet to discuss patients flagged and refer them to the IDVA upon their next attendance:

“Repeat attendances at emergency department as a result of the abuse will come up on the system … X number of times in before it’s a red flag, then goes straight to IDVAs.” (Location 1, Emergency Department Nurse)

“We meet all hospital staff concerned with domestic abuse every 1-2 months. Up until about year ago we never had that. I had all this information, but we didn’t really do much.” (Location 4, Emergency Department Nurse)

A third strategy was for hospital-wide domestic violence and abuse policies to clearly communicate the aim of the IDVA service and how to access it. However, healthcare professionals pointed out that there is no standardisation across NHS Trusts of policies about domestic violence and abuse and that policies were clearer and more well-known in some organisations than in others:

“Following disclosure, we follow a flowchart. Some staff probably don’t because they don’t want to do that, or don’t know it is there, or can’t be bothered. A lot of doctors just do their own thing.” (Location 4, Emergency Department Nurse)

Finally, healthcare professionals highlighted the importance of joined-up working with other services, agencies, and clinical teams, such as through discussion of cases at regular team meetings. They said that getting feedback on referred patients would be motivating and encourage a better working relationship with the IDVA:

“I’d like more knowledge of what happens next … I make an initial referral and never find out what happens next … doesn’t help motivate me to make referrals.” (Location 4, Consultant Psychiatrist)

As the above quotes illustrate, healthcare professionals valued close communication with the IDVAs and were keen for this communication to continue to improve. Thus, there was more work to be done to develop IDVA service models and this work would need cooperation between commissioners, data officers, as well as healthcare professionals and domestic violence and abuse organisations.

## Discussion

This paper reports the first qualitative study of staff views about a hospital-based IDVA intervention. It adds to the existing literature, which does not outline the value and problems associated with IDVA interventions.

Our findings showed that healthcare professionals thought the hospital-based IDVA service was important; that the service offered valuable ongoing training and support; that it improved healthcare professionals’ confidence identifying and responding to domestic violence and abuse; and that it helped survivors to receive immediate support. Since IDVAs could refer to other services and departments within the hospital, support was holistic and multi-disciplinary, and the service stopped survivors being lost along the referral pathway. As with previous research [22], healthcare professionals and IDVAs felt that the IDVA reached survivors with complex needs and multiple disadvantages, as well as other ‘hidden’ survivors—older people, those from higher-income households, and men. Reaching men was important because as earlier research has shown, healthcare professionals do not always realise the extent of male victimhood [14]. The statistical data from the quantitative aspect of our evaluation [27] largely mirrors interviewees’ views in this paper: the data showed that hospital IDVAs worked with survivors who were older (aged over 55) and from higher income households, although referrals for black, Asian, and minority ethnic people and lesbian, gay, bisexual and transgender people were low. The quantitative evaluation also confirmed that hospital IDVAs were more likely to engage with survivors at an earlier point in the abusive relationship, often when survivor and perpetrator were cohabiting, which suggests that the service provided opportunity for early intervention. The IDVA service helped healthcare professionals overcome barriers to asking about domestic violence and abuse, such as the worry that they would have no immediate support to offer if the patient disclosed. This barrier that has emerged in previous studies [11, 15].

Our findings echo the evaluations of domestic abuse nurse specialists [5–7]. These evaluations have shown that healthcare professionals valued receiving ongoing training and support (e.g. for managing the discomfort and distress identifying domestic violence and abuse) from the nurse and felt it boosted their confidence in asking about abuse. Healthcare professionals also appreciated the nurse’s expertise and ability to dedicate time to seeing patients—something they were unable to do themselves in busy clinics.

The perceived effectiveness of the IDVA service was dependent on many factors. As with previous interventions [14, 15], healthcare professionals pointed out that one-off training would be insufficient: training would need to be ongoing to change (potentially entrenched) attitudes and practices, to maintain learning, and to capture new staff. In the Identification and Referral to Improve Safety (*IRIS)* general practice programme [13], ongoing training of practice teams is integral, but is challenging given the competing training demands on primary care clinicians [30]. Moreover, although maternity services have tried to incorporate routine enquiry, provision of training for midwives is also inconsistent and poorly integrated [31].

Healthcare professionals in the current study suggested that medical curricula and postgraduate training should incorporate domestic violence and abuse training to entrench good practice among doctors from early in their careers. The UK’s National Institute for Health and Care Excellence guidelines in fact recommend that medical education should include teaching on domestic violence and abuse. There is scope to incorporate such training: an online survey completed by teaching leads at 25 of the 34 UK medical schools showed that 21 delivered some education around domestic violence and abuse. However, of these, 11 reported providing zero to two contact hours on the subject over a five-year degree. Three quarters felt provision of domestic violence and abuse teaching was inadequate or not enough [32]. Similar audits are needed on domestic violence and abuse teaching on UK nursing and other healthcare professional curricula.

Interviewees pointed out that the IDVA being ‘visible’ through having a dedicated physical space would remind healthcare professionals to use the service, which would in turn strengthen IDVAs’ and healthcare professionals’ relationships. Space would enable private meetings with patients which would enhance survivor safety, help maintain confidentiality, and make the IDVA feel like a legitimate part of the team. However, as with IDVAs in the *REACH* emergency department service [24], finding dedicated private space was difficult. In the domestic abuse nurse specialist project, the nurse’s integration in the team was facilitated by the fact that she had previously worked in the department [7].

One factor that helped to integrate the IDVA service was for healthcare professionals to be able to record disclosure of abuse in patients’ medical records and for IDVAs to have access to the records. Previous work has emphasised the value of electronic medical records prompting enquiry and enabling accurate recording of (suspected) domestic violence and abuse [33]. Drinkwater et al. [34] explored UK general practice staff views on medical records and found standardised codes for recording domestic violence and abuse were not always available. Moreover, local and national policies about how to record domestic violence and abuse varied widely. Clinicians furthermore worried that perpetrators might see the medical record e.g., if they see it in the clinic or if they request their child’s medical records, which could lead to an escalation of abuse, including towards children. Further discussion and training should focus on how hospitals can use medical records to improve referrals to domestic violence and abuse services.

Interviewees in the current study highlighted that hospital-wide strategies could help increase healthcare professionals’ awareness of, and referrals to, the IDVA service. Warren-Gash et al. [22] highlighted the importance of such strategies at the local level and spent time introducing the IDVA at meetings, distributing business cards, and adding information about the service to the staff intranet. Commissioners and hospital managers could use existing structures such as safeguarding training as an opportunity to make healthcare professionals aware of the IDVA service and to deliver domestic violence and abuse training. Healthcare professionals also said feedback about onward referrals would motivate them to use the service. Feedback is a central part of the *IRIS* primary care model [13]. An earlier evaluation of domestic abuse nurse specialists shows that these nurses, unlike external domestic violence and abuse agencies, were able to provide healthcare professionals with feedback, which healthcare professionals valued [7].

Our future work will explore how to support healthcare professionals who are survivors themselves and how personal experience might affect identifying and responding to patients. As one participant said, “People would find the IDVA in a corridor and tell them quite powerful stuff … quite lot of people felt uncomfortable because it brought up a lot for themselves.” In her work on nurse specialists, McGarry [5] similarly found that staff disclosed domestic violence and abuse after training and the nurse worked with the NHS Trust to devise staff support mechanisms.

### Funding and sustainability

In the UK, NHS England (part of the UK government’s Department of Health and Social Care) has a budget which it allocates to Clinical Commissioning Groups. These groups decide how to spend their budget according to ‘needs assessments’. They have a statutory responsibility for commissioning most NHS services, but IDVA services are not mandatory. NHS England, Public Health England (an executive agency of the Department), and local government can also commission services. Through demonstrating the value and effectiveness [27] of hospital-based IDVAs in this evaluation, we hope to show commissioners that they are worth funding.

Indeed, hospital-based IDVAs can save hospitals money. In Basu and Ratcliffe’s [23] emergency department-based hospital intervention, the NHS Trust funded IDVAs based on the cost-effectiveness of an earlier maternity-based IDVA service, which reduced hospital attendances and admissions. Safelives [28] recommend that two IDVAs per NHS Trust should be employed at minimum to ensure staff are not lone working, across a seven-day service, including evenings. This would equate to a spend of around £100,000 per NHS provider, or £15.7 million in total. A more detailed breakdown of the financial implications of the service is available in the quantitative evaluation [27].

Funding must also ensure the sustainability of IDVA services, as illustrated by the recent curtailing of the 5 year old *REACH* [24] emergency department hospital-based IDVA service due to funding cuts [35]. There are questions about the sustainability of the IDVA service evaluated here: IDVAs were often on short honorary contracts, which likely made it challenging for them to feel embedded in the NHS Trust and to provide support and ongoing training to the huge hospital community. Based on the findings of this study, funding from commissioners must include costs for ongoing training, physical space for the IDVA, integration of flagging in medical records, and for IDVAs’ contracts to be long enough to embed the service into the specific healthcare setting.

### Limitations

Our research has some limitations. To retain anonymity, we have been unable to provide some context for the intervention sites and no data exist on domestic violence and abuse prevalence for the communities surrounding the hospitals. Methodology-wise, the research was initially led by a busy domestic violence and abuse charity and there was not capacity to, for example, listen to interview recordings and double-check transcripts, to use respondent verification to strengthen trustworthiness, or to do longitudinal qualitative research.

## Conclusions

Our study has shown that healthcare professionals valued the hospital-based IDVA service and although the study does not evaluate effectiveness, it shows healthcare professionals saw it as an effective way of tackling domestic violence and abuse in a healthcare environment. IDVAs’ co-location in hospitals encouraged confidence among healthcare professionals about responding to domestic violence and abuse and meant survivors—including those hidden to other services—could get specialist support quickly. We recommend further work with commissioners to explore how to implement the service in a sustainable way and address the potential barriers to widespread implementation.

## Data Availability

The datasets generated and/or analysed during the current study are not publicly available because participant consent was not sought for this purpose. Data may be available from the corresponding author on reasonable request.

## Abbreviations

HIV: Human immunodeficiency viruses
IDVA: Independent domestic violence advisor
NHS: National Health Service
UK: United Kingdom
